# Proteomics approach to identify serum biomarkers associated with the progression of diabetes in Korean patients with abdominal obesity

**DOI:** 10.1371/journal.pone.0222032

**Published:** 2019-09-10

**Authors:** Sang Woo Kim, Jung-Won Choi, Jong Won Yun, In-Sung Chung, Ho Chan Cho, Seung-Eun Song, Seung-Soon Im, Dae-Kyu Song

**Affiliations:** 1 Institute for Bio-Medical Convergence, College of Medicine, Catholic Kwandong University, Gangneung-si, Gangwon-do, South Korea; 2 Catholic Kwandong University, International St. Mary’s Hospital, Incheon Metropolitan City, South Korea; 3 Department of Biotechnology, Daegu University, Kyungsan, Kyungbuk, South Korea; 4 Division of Occupational and Environmental Medicine and Department of Preventive Medicine, Keimyung, University School of Medicine, Daegu, South Korea; 5 Department of Internal Medicine, Keimyung, University School of Medicine, Daegu, South Korea; 6 Department of Physiology and Obesity-mediated Disease Research Center, Keimyung, University School of Medicine, Daegu, South Korea; University of Hawai'i at Manoa College of Tropical Agriculture and Human Resources, UNITED STATES

## Abstract

Type 2 diabetes is a metabolic disease with a group of metabolic derangements and inflammatory reactants in the serum. Despite the substantial public health implications, markers of diabetes progression with abdominal obesity are still needed to facilitate early detection and treatment. In this study, we performed a proteomic approach to identify differential target proteins underlying diabetes progression in patients with abdominal obesity. Proteomic differences were investigated in the serum of controls and patients with prediabetes or diabetes with or without abdominal obesity by 2-DE combined with MALDI-TOF-MS. Proteomics data were validated by western blot analyses and major protein-protein interactions were assessed using a network analysis with String database. Among 245 matched protein spots, 36 exhibited marked differences in normal patients with abdominal obesity, prediabetes, and diabetes compared to levels in normal patients without abdominal obesity. Seven (Alpha-1-antichymotrypsin, Alpha-1-antitrypsin, Apolipoprotein A-I, haptoglobin, retinol-binding protein 4, transthyretin, and zinc-alpha2-glycoprotein) of these spots exhibited significant differences between normal and prediabetes/diabetes patients. After a network analysis, functional annotation using Gene Ontology indicated that most of the identified proteins were involved in lipid transport, lipid localization, and the regulation of serum lipoprotein particle levels. Our results indicated that variation in the levels of these identified protein biomarkers has been reported in normal, prediabetes and diabetic Assessment of the levels of these biomarkers may contribute to the development of biomarkers for not only early diagnosis but also in prognosis of diabetes mellitus type 2.

## Introduction

Obesity and type 2 diabetes are global healthcare problems that threaten to reach epidemic proportions in many countries [1–3]. Serum lipid and lipoprotein abnormalities are common in both insulin-dependent and non-insulin-dependent diabetes mellitus [4]. The concentrations of inflammatory mediators in the serum are elevated in the insulin-resistant states of obesity and type 2 diabetes [5, 6]. Increases in inflammatory factors derived from adipose tissues predict the future development of obesity and diabetes and accordingly have been a focus of research [7]. Several studies have demonstrated that type 2 diabetes is associated with increases in the concentrations of inflammatory reactants in serum [8, 9]. Increased concentrations of tumor necrosis factor (TNF)-α and interleukin (IL)-6 associated with obesity and type 2 diabetes might interfere with the anti-inflammatory effects of insulin, which can promote inflammation [10]. When diabetes develops, numerous inflammatory cytokines can be used to detect insulin resistance and predict fasting serum glucose [11].

Serum proteome analyses can be used to identify diagnostic or prognostic biomarkers and provide insight into the mechanisms underlying disease development and progression [12–15]. A panel of candidate biomarkers is typically needed to improve diagnostic efficacy, since single protein marker often does not fully predict a condition with significant clinical value [16, 17]. As an analytical technique for the quantification of disease-associated alterations, two-dimensional electrophoresis (2-DE) combined with mass spectrometry is often applied to complex biological samples owing to its unique ability to simultaneously resolve hundreds to thousands of proteoforms in a single analytical run [18].

In this study, we used a proteomics approach to identify target proteins involved in diabetes progression in patients with prediabetes and diabetes with respect to their obesity (or overweight) state. We provide the first evidence that protein biomarkers could play a key role in the development of diabetes in abdominal obesity. These proteins are strong candidate biomarkers for clinical prediction and applications.

## Materials and methods

### Study subjects and design

This study was approved by the Institutional Review Board of Keimyung University Dongsan Medical Centre in Korea (2015-03-010) and informed consent was obtained from each subject. Surveys and clinical examinations were performed for 36 male participants who visited the Keimyung University Dongsan Medical Centre for health and medical examination from May 2016 to April 2017. The subjects were divided into six groups based on fasting blood glucose and glycated hemoglobin (HbA1c) levels (indicators of the severity of diabetes) and the waist circumference/hip circumference ratio (WHR; a measure of abdominal obesity) as follows: patients with type 2 diabetes (DM; *n* = 6) if FBS > 126 mg/dL or HbA1c > 8.0 mmol/L, pre-diabetes (pre-DM, *n* = 6) if FBS ≈ 110 ± 5 mg/dL and HbA1c ≈ 6.0 ± 0.2 mmol/L, and non-diabetic control (*n* = 6) if FBS < 100 mg/dL and HbA1c < 5.7 mmol/L. The optimal WHR cut-off for abdominal obesity is 0.9 in men. Patients with similar body mass index (BMI) were grouped into each group.

### Preparation of serum samples from donors/patients

Serum samples were collected from 36 patients (6 donors per group, six total groups) at various time points after obtaining written informed consent, as described before. Basic information for the 36 patients (normal, *n* = 6; normal with abdominal obesity, *n* = 6; pre-diabetes, *n* = 6; pre-diabetes with abdominal obesity, *n* = 6; diabetes, *n* = 6; diabetes with abdominal obesity, *n* = 6) is shown in Table 1.

**Table 1:** Physical characteristics of patients with normal and prediabetes/diabetes groups with and without abdominal obesity.

Variable	Group 1 ^a)^ (*n* = 6)	Group 2 (*n* = 6)	Group 3 (*n* = 6)	Group 4 (*n* = 6)	Group 5 (*n* = 6)	Group 6 (*n* = 6)
Age (years)	40.83 ± 2.56	44.67 ± 8.36	45.83 ± 3.66	45.00 ± 2.75	48.17 ± 7.55	48.50 ± 7.89
BMI (kg/m^2^) ^**b)**^	20.93 ± 0.54	24.20 ± 0.59	24.73 ± 1.68	27.10 ± 0.52	23.97 ± 1.61	27.49 ± 0.97
HbA1C (mmol/L)	5.03 ± 0.27	5.18 ± 0.23	5.85 ± 0.17	5.871 ± 0.12	8.20 ± 1.47	8.02 ± 1.74
Waist circumference (cm)	76.43 ± 1.96	91.52 ± 1.23	85.30 ± 3.45	92.83 ± 2.34	83.45 ± 5.38	97.17 ± 6.28
Hip circumference (cm)	92.18 ± 1.83	97.15 ± 1.13	97.68 ± 4.25	102.98 ± 1.48	96.30 ± 3.86	101.67 ± 1.04
WHR ^**c)**^	0.83 ± 0.02	0.94 ± 0.01	0.87 ± 0.03	0.90 ± 0.03	0.87 ± 0.05	0.96 ± 0.06
Body fat (%)	15.30 ± 2.02	26.53 ± 2.89	21.63 ± 2.67	25.18 ± 3.91	20.88 ± 4.02	27.27 ± 6.21
Body fat mass (kg)	9.58 ± 1.26	19.25 ± 2.41	15.58 ± 2.96	20.50 ± 2.45	14.42 ± 3.15	22.93 ± 5.87
Skeletal muscle mass (kg)	29.87 ± 2.63	29.93 ± 1.32	31.62 ± 3.63	34.93 ± 3.85	30.77 ± 4.01	34.23 ± 3.07
Total cholesterol	182.33 ± 10.11	205.17 ± 29.16	224.17 ± 40.76	221.00 ± 20.59	180.17 ± 44.58	180.00 ± 14.74
AST	19.17 ± 3.37	27.17 ± 14.80	18.00 ± 3.39	24.00 ± 3.16	20.80 ± 5.93	31.67 ± 18.41
ALT	12.33 ± 6.19	35.33 ± 43.18	17.40 ± 7.27	31.80 ± 4.44	16.66 ± 6.27	43.50 ± 50.29
TG	113.75 ± 46.19	183.52 ± 87.89	224.23 ± 167.48	214.50 ± 94.48	215.87 ± 121.33	150.17 ± 82.15
HDL	53.08 ± 15.32	43.63 ± 11.82	43.25 ± 2.17	40.70 ± 5.80	40.43 ± 9.50	43.67 ± 6.84
LDL	107.67 ± 17.28	120.67 ± 28.92	143.00 ± 31.58	142.00 ± 19.85	101.00 ± 50.53	117.40 ± 32.52

a) Patients were assigned to six groups according to their BMI as well as blood FBS and HbA1C levels: normal (Group 1), normal with abdominal obesity (Group 2), pre-diabetes (Group 3); pre-diabetes with abdominal obesity (Group 4), diabetes (Group 5), diabetes with abdominal obesity (Group 6).

b) BMI, body mass index.

c) WHR = waist circumference/hip circumference ratio.

### 2-DE analysis

2-DE was performed in triplicate using serum samples (150 μg per gel) from each donor. Immobilized pH gradient (IPG)-isoelectric focusing (IEF) of serum samples was performed at pH 4–7 with 18-cm IPG DryStrips (GE Healthcare, Buckinghamshire, UK) using an Ettan^TM^IPGphor^TM^ 3 system (GE Healthcare) following the protocol recommended by the manufacturer. The IPG strips were passively rehydrated for 12 h in strip holders with 340 μL of DeStreak Rehydration Solution (GE Healthcare), which included 30 μg of protein in each sample. IEF was executed using the advanced mode protocol as follows: 1 h at 500 V, 3 h at 1,000 V, and 6 h at 7,000 V, followed by holding at 7,000 V until reaching 115 KVh. The gel strips were then placed onto a 12% polyacrylamide gel for resolution along the second dimension, which was performed using an Etthan DALTsix system. Fractionation was performed according to the manufacturer’s instructions. A total of six gels, i.e., three gels containing separated proteins per group, were visualized using silver staining and were used for image analysis and peptide mass fingerprinting (PMF) [19].

### Image acquisition and data analysis

Gel imaging was performed using a UMAX PowerLook 1120 system (Maxium Technologies, Akron, OH, USA) and modified ImageMaster 2-D V4.95 (GE Healthcare). The detected spots on all gels were matched to those on the reference gel, which was selected from the gels of the normoxic cell group. Relative optical densities and relative volumes were calculated to correct for differences in in-gel staining. The intensity of each spot was processed using background subtraction and total spot volume normalization, and the resulting spot volumes (expressed as percentages) were used for comparisons between groups.

### Protein identification

Protein spots were excised, digested with trypsin (Promega, Madison, WI, USA), mixed with α-cyano-4-hydroxycinnamic acid (Sigma-Aldrich, St. Louis, MO, USA) in 50% acetonitrile/0.1% trifluoroacetic acid, and used for MALDI-TOF (Microflex LRF 20; Bruker Daltonics, Billerica, MA, USA) as described by Fernandez *et al*. [20]. Spectra were collected from 300 shots per spectrum over an *m/z* range of 600–3000 and calibrated using a two-point internal calibration of trypsin auto-digested peaks (*m/z* 842.5099, 2211.1046). The peak list was generated using Flex Analysis 3.0. The thresholds for peak-picking were 500 for the minimum resolution of monoisotopic mass and 5 for S/N. The search program MASCOT, developed by Matrix Science (http://www.matrixscience.com), was used, and the MASCOT probability-based MOWSE (molecular weight search) score was calculated for PMF. For the database search, the following parameters were used: trypsin included as the cleaving enzyme, a maximum of one missed cleavage, iodoacetamide (Cys) as a complete modification, oxidation (Met) as a partial modification, monoisotopic masses, and a mass tolerance of ±0.1 Da. The PMF acceptance criteria were based on probability scoring as follows: -10*Log (*P*), where *P* is the probability that the observed match is a random event, and a score of greater than 62 was significant (*p* < 0.05).

### Enzyme-linked immunosorbent assay (ELISA)

Interferon (IFN)-γ and TNF-α levels in serum samples were quantitatively measured using the Human IFN-γ ELISA Kit (Abcam; Cambridge, UK) and Human TNF-α ELISA Kit (Abcam), following the manufacturer’s instructions.

### Immunoblot analysis

Differential expression of each protein of interest was evaluated by dilution in 2× sample buffer (50 mM Tris (pH 6.8), 2% SDS, 10% glycerol, 0.1% bromophenol blue, and 5% β-mercaptoethanol) and heated for 1 min at 100°C. After SDS-polyacrylamide gel electrophoresis (PAGE), proteins were transferred to a polyvinylidene difluoride (Santa Cruz Biotechnology, Santa Cruz, CA, USA) membranes and blocked for 30 min with TBS (Tris-buffered saline)-T buffer (10 mM Tris-HCl, 150 mM NaCl, and 0.1% Tween 20) containing 5% skim milk. The membranes were then washed in five changes of TBS-T buffer, followed by incubation overnight at 4°C with the appropriate primary antibody (Santa Cruz Biotechnology) diluted 1:1000 in TBS-T buffer containing 5% bovine serum albumin (AMRESCO, Solon, OH, USA) and 0.02% sodium azide (Sigma-Aldrich). After five washes, the membranes were incubated again for 30 min with horseradish peroxidase (HRP)-conjugated anti-goat IgG, anti-mouse IgG, or anti-rabbit IgG secondary antibody (Santa Cruz Biotechnology) diluted 1:2000 in TBS-T buffer containing 5% skim milk. After washing the membranes five times with TBS-T buffer, they were visualized using an enhanced chemiluminescence system (ECL, Western Blotting Detection Kit; GE Healthcare), and band intensities were quantified using ImageJ (NIH, Bethesda, MD, USA).

### Network analysis

For gene and protein network analyses, differentially expressed proteins identified in the proteomic analysis were analyzed using String v10.5 (http://string-db.org) [21]. Associations between differentially expressed genes and proteins with broadly defined molecular networks were combined and visualized using the String database. Using the web interface, interactions between identified genes/proteins and their interacting partner proteins identified in this study were predicted.

### Statistical analysis of experimental data

All results were evaluated by one-way analysis of variance (ANOVA) using Statistical Package of Social Science (SPSS, version 14.0K). Results are expressed as means ± SEM. Group means were significantly different at *p* < 0.05, as determined by the protected least-significant difference (LSD) test when an ANOVA followed by Tukey’s honest significant difference test.

## Results

### Characteristics of patients

Thirty-six patients were assigned to six groups: Group 1 (normal, *n* = 6), Group 2 (normal with abdominal obesity, *n* = 6), Group 3 (pre-diabetes, *n* = 6), Group 4 (pre-diabetes with abdominal obesity, *n* = 6), Group 5 (diabetes, *n* = 6), and Group 6 (diabetes with abdominal obesity, *n* = 6). The characteristics of the six groups are presented in Table 1. The highest concentrations of total cholesterol and triglycerides were observed in Group 3, and the AST/ALT ratio in the serum was higher in patients with abdominal obesity (Groups 2, 4, and 6) than in those without abdominal obesity (Groups 1, 3, and 5).

### Identification of differentially expressed proteins in prediabetes/diabetes compared with normal serum

**Fig 1:**
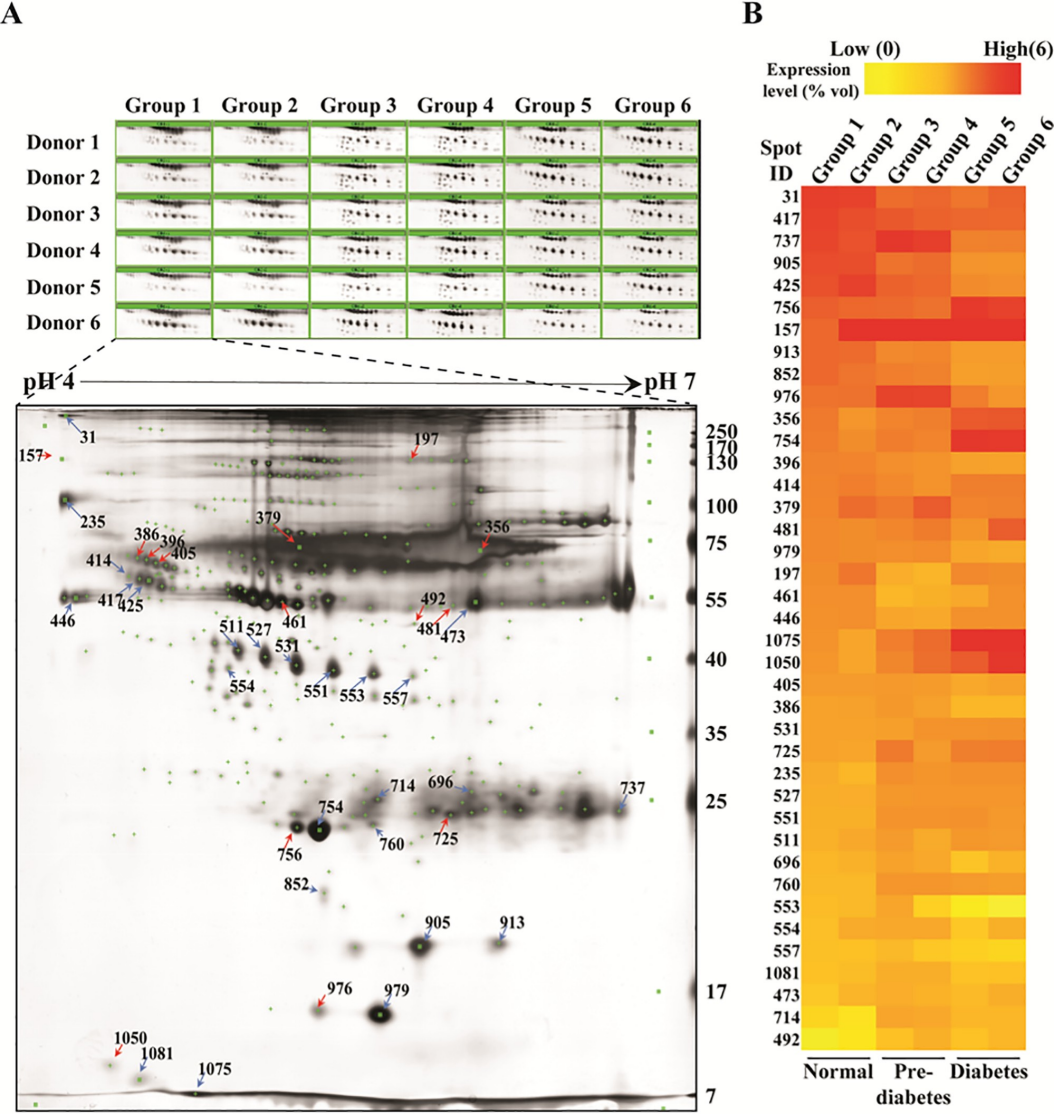
Representative silver-stained two-dimensional electrophoresis (2-DE) images for serum samples from patients with prediabetes/diabetes compared with healthy donors. (A) Differentially expressed spots are indicated with arrows (Red, up-regulated; Blue, down regulated) in the 2-DE image, (B) comparison of the regulatory patterns of serum proteins in the six groups. Group 1 = normal, Group 2 = normal with abdominal obesity, Group 3 = pre-diabetes, Group 4 = pre-diabetes with abdominal obesity, Group 5 = diabetes, Group 6 = diabetes with abdominal obesity. See Table 1 for patient details.

To investigate the differential expression of proteins in the serum among groups, we performed 2-DE-based proteomic experiments. Proteins were separated by 2-DE using pH 4–7 IEF strips for the first dimension and 12% (w/v) SDS-PAGE for the second dimension. Among 245 matched spots, 25 were highly up-regulated and 11 were significantly down-regulated in normal patients with abdominal obesity (Group 2), prediabetes (Groups 3 and 4), and diabetes (Groups 5 and 6) compared to levels in normal patients without abdominal obesity (Group 1), ranging in mass from 6 to 240 kDa between pH 4 and 7 (Fig 1A and 1B). According to MALDI-TOF identification, the proteins that were more highly expressed in normal patients with abdominal obesity (Group 2), prediabetes (Groups 3 and 4), and diabetes (Groups 5 and 6) than in normal patients without abdominal obesity (Group 1) included serpin peptidase inhibitor A1 (AAT/SERPINA1), haptoglobin protein (HP), zinc-alpha2-glycoprotein (ZAG), apolipoprotein A-1 (APOA1), and retinol binding protein 4 (RBP4) (Table 2). In contrast, growth-inhibiting protein 25 (GIG25/AACT/SERPINA3), albumin (ALB), and transthyretin (TTR) were expressed at lower levels in normal patients with abdominal obesity (Group 2), prediabetes (Groups 3 and 4), and diabetes (Groups 5 and 6) than in normal patients (Group 1).

**Table 2:** List of identified proteins showing differential expression between normal and prediabetes/diabetes groups.

Protein ID ^a)^	Gene name	Description	Acc. no. ^b)^	Nominal mass (*Mr*) ^c)^	Calculated PI	Score ^d)^
31		ND ^e)^				
157	*C1NH*	C1-inhibitor, partial	AAA51848.1	32745	8.85	72
197	*ALB*	PRO2619	AAG35503.1	58513	5.96	114
235	*SERPING1*	serpin peptidase inhibitor, clade G (C1 inhibitor), member 1, (angioedema, hereditary), isoform CRA_a	EAW73762.1	45526	6.01	68
356	*ALB*	albumin, isoform CRA_h	EAX05666.1	70564	5.92	223
379	*ALB*	PRO2619	AAG35503.1	58513	5.96	221
386	*GIG25*	growth-inhibiting protein 25	AAT08029.1	32857	5.71	165
396	*GIG25*	growth-inhibiting protein 25	AAT08029.1	32857	5.71	110
405	*GIG25*	growth-inhibiting protein 25	AAT08029.1	32857	5.71	90
414		ND				
417	*AHSG*	alpha-2-HS-glycoprotein isoform 4 preproprotein	NP_001341502.1	37010	5.41	78
425	*AHSG*	alpha-2-HS-glycoprotein, isoform CRA_a	EAW78187.1	39970	5.43	87
446	*SERPINA1*	Serpin peptidase inhibitor, clade A (alpha-1 antiproteinase, antitrypsin), member 1	AAH11991.1	46864	5.36	139
461	*SERPINA1*	Serpin peptidase inhibitor, clade A (alpha-1 antiproteinase, antitrypsin), member 1	AAH15642.1	46850	5.37	104
473	*DKFZp686N02209*	hypothetical protein	CAD98026.1	53503	8.74	146
481	*ALB*	albumin, isoform CRA_k	EAX05669.1	48568	5.97	87
492	*ALB*	PRO2675	AAF69644.1	33466	6.14	101
511	*HP*	HP protein	AAH70299.1	31647	8.48	141
527	*HP*	HP protein	AAH70299.1	31647	8.48	110
531	*HP*	HP protein	AAH70299.1	31647	8.48	150
551	*HP*	HP protein	AAH70299.1	31647	8.48	156
553	*HP*	HP protein	AAH70299.1	31647	8.48	159
554	*ZAG*	zinc-alpha2-glycoprotein precursor	BAA14417.1	34079	5.57	173
557	*HP*	HP protein	AAH70299.1	31647	8.48	82
696		ND				
714	*APCS*	serum amyloid P-component precursor	NP_001630.1	25485	6.10	104
725		IgG kappa chain, partial	BAA37169.1	23690	6.92	112
737		IgG kappa chain, partial	BAA37169.1	23690	6.92	114
754	*APOA1*	apolipoprotein A-I isoform 1 preproprotein	NP_000030.1	30759	5.56	230
756	*APOA1*	apolipoprotein A-I isoform 1 preproprotein	NP_000030.1	30759	5.56	179
760	*APOA1*	apolipoprotein A-I isoform 1 preproprotein	NP_000030.1	30759	5.56	192
852	*RBP4*	Retinol binding protein 4, plasma	AAH20633.1	23371	5.76	140
905		ND				
913	*HP*	haptoglobin, partial	ALX40934.1	13744	6.10	76
976	*TTR*	Transthyretin	ABI63345.1	20300	5.16	76
979	*TTR*	transthyretin precursor	NP_000362.1	15991	5.52	137
1050		ND				
1075	*APOA2*	apolipoprotein A-II, isoform CRA_d, partial	EAW52622.1	10630	9.10	112
1081		unnamed protein product	BAG51195.1	15846	6.06	77

a) Protein IDs correspond to the numbers in 2DE images in Fig 1A.

b) Acc. no. is the NCBInr database accession number.

c) The nominal mass is the integer mass of the most abundant naturally occurring stable isotope of an element. The nominal mass of a molecule is the sum of the nominal masses of the elements in its empirical formula.

d) Protein score is -10 × Log (P), where P is the probability that the observed match is a random event. Protein scores of greater than 62 are significant (*p* < 0.05).

e) ND: not detected.

### Validation of differentially expressed serum proteins in prediabetes/diabetes compared with normal serum

**Fig 2:**
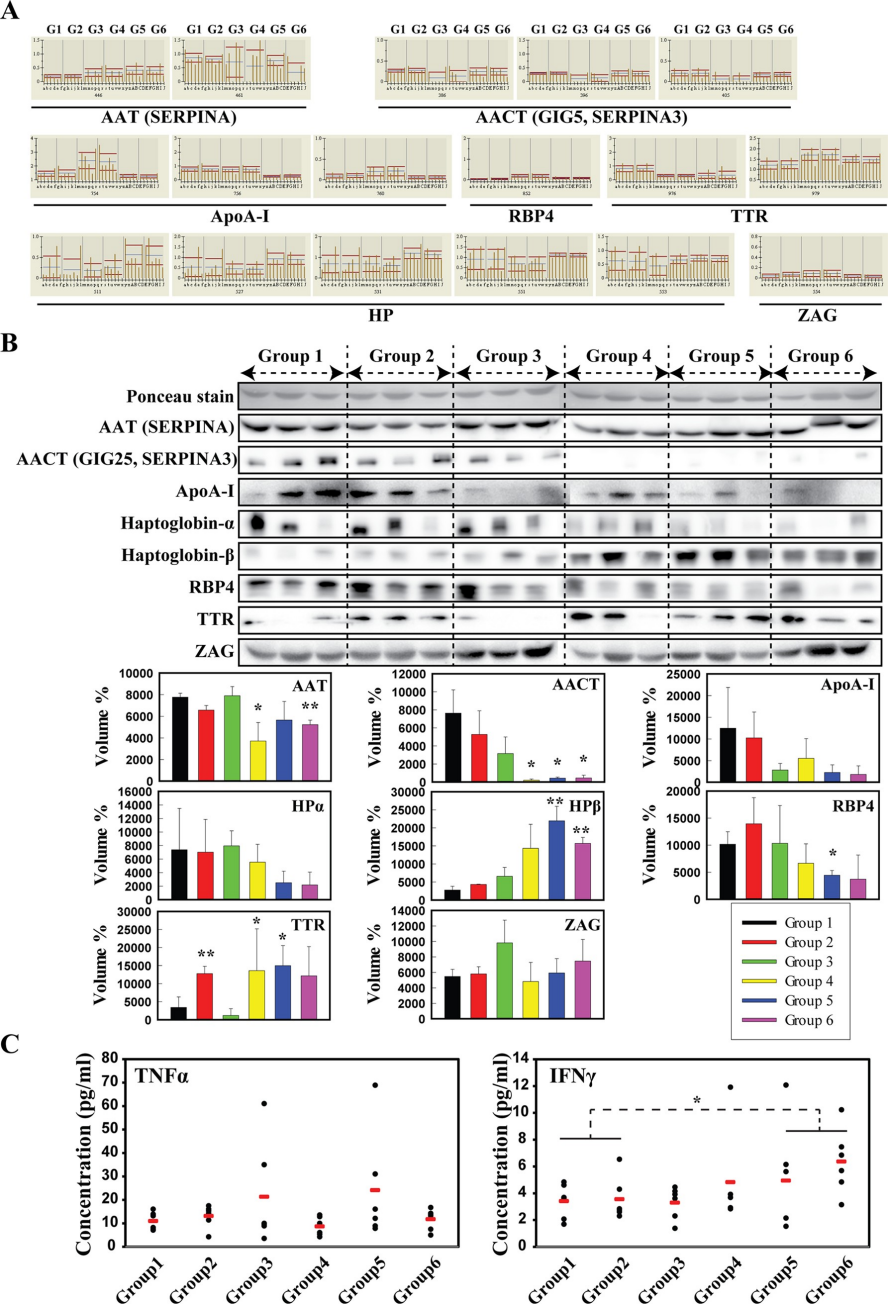
Differentially regulated proteins in the serum of patients with prediabetes/diabetes compared with healthy donors. (A) Normalized graphs of 2-DE spots showing relative upward/downward trends in regulation (G1–G6 indicate patient groups), (B) validation of target proteins linked to abdominal obesity and diabetes. Significant differences among groups (where, each Group was compared to Group 1) were determined by ANOVA, *p < 0.05 or **p < 0.01. (C) Expression levels of pro-inflammatory cytokine genes, TNFα and IFNγ in serum of patients with prediabetes/diabetes compared with healthy donors. Significant differences between normal (Groups 1 and 2) and prediabetes (Groups 3 and 4)/diabetes (Groups 5 and 6) groups were determined by ANOVA; *p < 0.05.

Seven (AACT, AAT, ApoA-I, HP, RBP4, TTR, and ZAG) proteins among identified proteins (Table 2) that were clearly related to the disease according to the proteomics analysis were selected for further validation (Fig 2A). Using western blot analyses, we examined the expression of these seven proteins in all groups and found lower AAT and AACT expression in Groups 4, 5, and 6 than in Group 1, and no difference between the normal groups (Groups 1 and 2) (Fig 2B). Two of ApoA-I spots and RBP4 spot were expressed higher in pre-diabetic status (Groups 3 and 4), while expression level of all spots decreased in diabetic groups (Groups 5 and 6). In addition, HPα spots were down-regulated in pre-diabetic status (Groups 3 and 4), while three (Spot ID: 531, 527, 551) of five spots expression was higher in diabetic patients (Groups 5 and 6) than in Group 1, and there were no differences between the normal groups (Groups 1 and 2). TTR expression was significantly higher in Groups 2 and 5 than in Group 1, and ZAG did not differ significantly among groups (Fig 2B). As no reliable internal control protein was available for western blot analyses of serum samples, a loading control sample was generated from all groups in equal volumes for each gel and Ponceau S staining was used to determine the amount of protein loaded.

Next, we explored whether inflammatory mediators enable to predict the development of type 2 diabetes as obesity and diabetes are in an inflammatory state in most cases. Several lines of evidence suggest that interferon-γ (IFNγ) and tumor necrosis factor-α (TNFα) levels are markedly increased in obesity [22–25]. These studies have suggested that the presence of inflammation predicts the development of type 2 diabetes. In the current study, IFNγ levels tended to be higher in patients with diabetes (Groups 5 and 6) than in normal individuals (Groups 1 and 2). However, unexpectedly, there were no significant differences in concentrations of TNFα among the six groups (Fig 2C).

### Functional partners predicted in a network analysis

**Fig 3:**
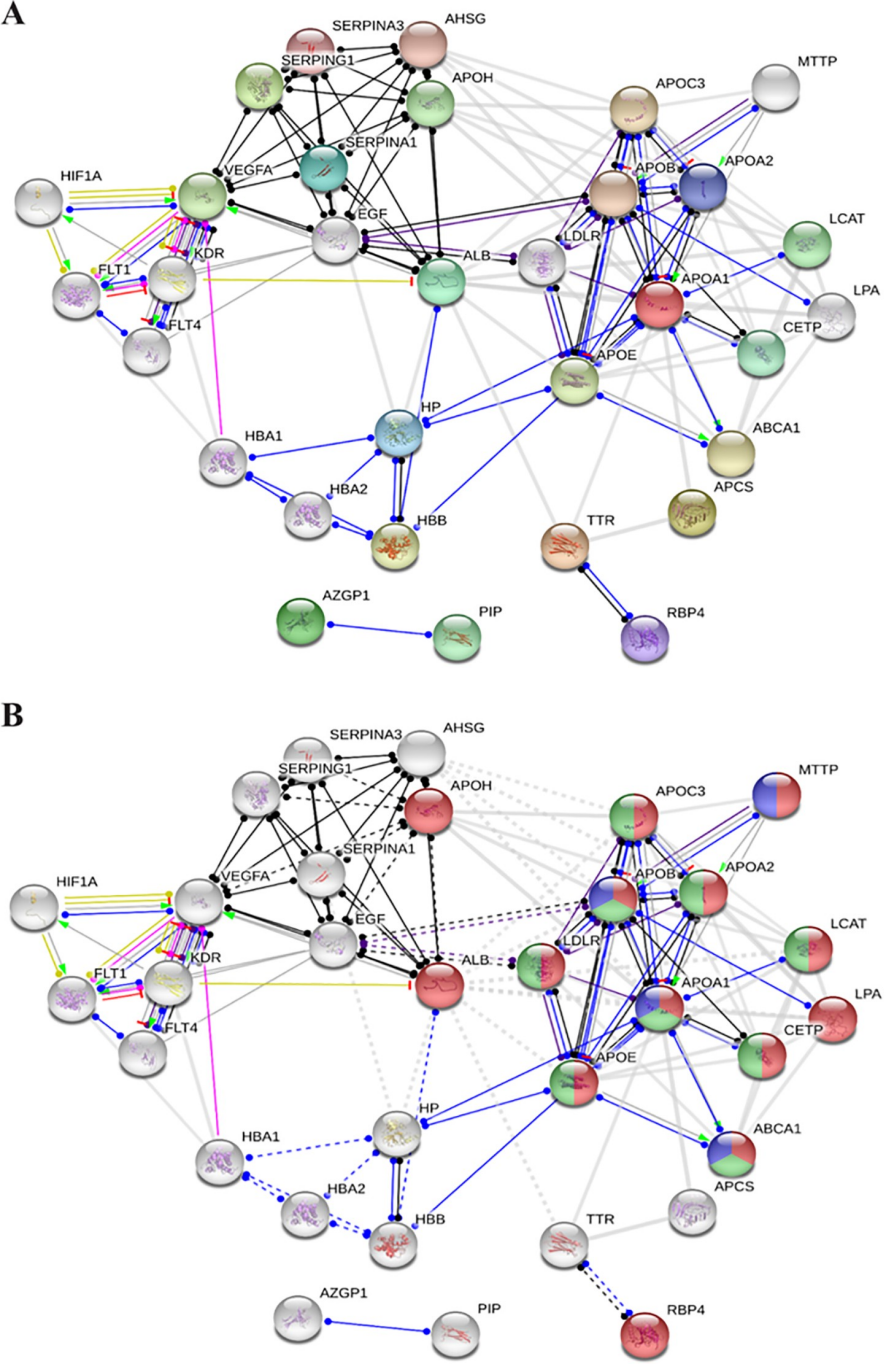
Networks generated by the String database analysis using up- and down-regulated proteins (A) and functional classification of the proteins in the network, where proteins related to lipid transport (red), regulation of plasma lipoprotein particle levels (green), and fat digestion/absorption (blue) are highlighted (B).

Based on the networks generated by the String database analysis using the identified proteins (AHSG, ALB, APCS, APOA1, APOA2, HP, RBP4, C1NH/SERPING1, SERPINA1, GIG25/SERPINA3, TTR, and ZAG) in each group, interactions were determined *in silico* (Fig 3A).Elements are listed according to enriched biological processes, molecular function, cellular components, and Kyoto Encyclopedia of Genes and Genomes (KEGG) pathways (Table 3). Target proteins are shown according to their functional classification in Fig 3B, i.e., they are marked in red (lipid transport), green (regulation of plasma lipoprotein particle levels), and blue (fat digestion and absorption).

**Table 3:** Summary of the functional classification of proteins in the network analysis.

	**Biological Process (GO)**		
*Pathway ID*	*Pathway description*	*Count in gene set*	*FDR* ^a)^
GO:0006869	Lipid transport	14	1.38e-15
GO:0010876	Lipid localization	14	2.74e-15
GO:0030301	Cholesterol transport	9	4.76e-14
GO:0097006	Regulation of plasma lipoprotein particle levels	9	4.76e-14
GO:0015850	Organic hydroxyl compound transport	11	1.17e-13
			(*more…*)
	**Molecular Function (GO)**		
GO:0017127	Cholesterol transporter activity	6	2.9e-10
GO:0005319	Lipid transporter activity	8	9.29e-10
GO:0015485	Cholesterol binding	6	2.44e-08
GO:0005515	Protein binding	24	4.84e-08
GO:0043178	Alcohol binding	7	4.84e-08
			(*more…*)
	**Cellular Component (GO)**		
GO:0005615	Extracellular space	26	6.78e-24
GO:0060205	Cytoplasmic membrane bounded vesicle lumen	12	1.17e-18
GO:0034358	Plasma lipoprotein particle	10	2.03e-18
GO:0072562	Blood microparticle	11	1.17e-15
GO:0071682	Endocytic vesicle lumen	7	4.46e-14
			(*more…*)
	**KEGG Pathways**		
04975	Fat digestion and absorption	4	0.000112
04104	Ras signaling pathway	5	0.00102
04015	Rap1 signaling pathway	5	0.00102
04066	HIF-1 signaling pathway	4	0.00102
04510	Focal adhesion	5	0.00102
			(*more…*)

a) FDR, false discovery rate.

## Discussion

In this study, we identified 36 differentially expressed protein spots among 245 total matched spots in serum from Korean patients with pre-diabetes/diabetes compared with healthy donors. We further discovered marked changes in prediabetes/diabetes-related proteins (AACT/SERPINA3, AAT/SERPINA1, ApoA-I, HP, RBP4, TTR, and ZAG). Seven proteins showing a clear trend related to the disease were further validated by western blotting. Moreover, we found that IFNγ levels tended to increase in diabetes with abdominal obesity.

A recent study demonstrated that dysfunctions of serine protease inhibitor (SERPIN) A1 and A3 were associated with liver disease [26]. To identify early diagnostic markers of diabetes, serum proteomics were performed to evaluate the KK-A^y^ mouse model of type 2 diabetes and identified high levels of SERPINA3 in the prediabetic state [26]. Contrary to the results of animal studies, SERPINA3 was significantly decreased in patients with prediabetes/diabetes in our study.

Transthyretin (TTR) exists both in a tetrameric and a monomeric form [27, 28]. Plasma transthyretin (TTR) is a plasma protein that circulates bound to retinol-binding protein 4 (RBP4) and is a potential indicator of the protein nutritional status [29]. The high expression of TTR in the dorsomedial hypothalamus of rats with exercise-induced anorexia suggests that central TTR may also play a functional role in modulating food intake and energy balance [30]. Methionine oxidized TTR represented a potential biomarker for type 2 diabetes. And TTR has received much more attention due to trends towards misfolding and aggregation disease [31]. Accumulated oxidized proteins appear in degenerative diseases and might contribute to the development of type 2 diabetes [32]. We also found that TTR was related to obesity and further validated its elevation in patients with obesity (overweight), indicating that this protein is closely associated with obesity and diabetes (Fig 2B).

ZAG plays a role in the pathogenesis of insulin resistance in newly diagnosed type 2 diabetes [33]. ZAG is a potential new adipose tissue protein factor that may be involved in the modulation of lipolysis in adipocytes [34]. According to our previous study, the serum protein levels of fetuin-B and ZAG are significantly elevated in obesity-resistant rats exposed to a high-fat diet [35]. In this study, our proteomic results showed that ZAG is related to the overweight status, but western blotting did not support significant differences among groups.

Over the past several decades, haptoglobin (HP) polymorphism has been linked to complications arising from diabetes [36]. HP is an acute-phase protein that eliminates free hemoglobin and the neutralization of oxidative damage. An interesting study showed that obesity modulates the expression of HP in white adipose tissue via TNFα [37]. HP forms a strong covalent complex with hemoglobin and is removed through the reticuloendothelial system and receptor-mediated endocytosis via CD163 on multiple cells and tissue macrophages. The expression of CD163 by these cells is induced by inflammation and the release of cytokines, such as IL-1 and IL-6. In particular, IL-6 has an important function in the stimulation of HP production and in the regulation of CD163 expression on the cell surface. CD163 is downregulated by TNFα, IL-4, and IFNγ, and IFNγ modulates important functions in adipocytes and is a central regulator of macrophage function [22]. In the current study, the TNF-α concentration in serum tended to be elevated in diabetes with abdominal obesity (Group 3 and Group 5), but there were no significant differences among the six groups. However, IFNγ levels tended to increase in diabetes with abdominal obesity and were significantly increased in Group 6. Collectively, serum HP may constitute a novel marker of adiposity in the acute phase in response to inflammation in humans, and adipose tissue is likely to contribute to its levels [38].

Obesity-induced inflammatory reactants are expressed in adipose tissue macrophages and are important mediators of insulin resistance in obesity. Crook et al. [8] and Pickup et al. [9] first proposed that type 2 diabetes was also an inflammatory condition characterized by elevated concentrations of acute phase inflammatory reactants in the serum. We observed that increased concentrations of TNFα and IFNγ, associated with obesity and diabetes, might interfere with the anti-inflammatory effect of insulin, which in turn might promote inflammation [10]. We suggest here that candidate biomarkers for early detection between normal patients with abdominal obesity, prediabetes, and diabetes and normal patients without abdominal obesity. And then, we further investigated relative changes in candidate biomarkers by Western blot analysis. However, we did not examine large-scale study of diabetes/obesity patients to identify target proteins involved in diabetes progression in patients with prediabetes and diabetes with respect to their obesity (or overweight) state, which remains one of the limitations of this study. Further studies using more patients groups will be helpful to identify candidate biomarkers for early detection of diabetes. Consequently, we first report here that HPβ and TTR may act as a protein biomarker that is specific for diabetes progression in overweight patients. Our results might provide a basis for the development of candidate biomarkers for the early diagnosis of pre-diabetes and/or diabetes status.
